# DHA supplementation and pregnancy complications

**DOI:** 10.1186/s12967-023-04239-8

**Published:** 2023-06-17

**Authors:** Yi Jiang, Yuting Chen, Lijie Wei, Huiting Zhang, Jingyi Zhang, Xuan Zhou, Shenglan Zhu, Yuanyuan Du, Rui Su, Chenyun Fang, Wencheng Ding, Ling Feng

**Affiliations:** 1 Department of Obstetrics and Gynecology, Tongji Hospital, Tongji Medical College, Huazhong University of Science and Technology, No. 1095, Jiefang Avenue, Wuhan, 430030 Hubei China; 2 Department of Obstetrics and Gynecology Ultrasound, Zhongnan Hospital of Wuhan University, Wuhan, 430071 China

**Keywords:** Docosahexaenoic acid, Pregnancy, Preeclampsia, Gestational diabetes mellitus, Preterm birth, Intrauterine growth restriction, Postpartum depression, Neuroprotection

## Abstract

Docosahexaenoic acid (DHA) supplementation is recommended for women during pregnancy because of its neurological, visual, and cognitive effects. Previous studies have suggested that DHA supplementation during pregnancy may prevent and treat certain pregnancy complications. However, there are contradictions in the current related studies, and the specific mechanism by which DHA acts remains unclear. This review summarizes the research on the relationship between DHA intake during pregnancy and preeclampsia, gestational diabetes mellitus, preterm birth, intrauterine growth restriction, and postpartum depression. Furthermore, we explore the impact of DHA intake during pregnancy on the prediction, prevention, and treatment of pregnancy complications as well as its impact on offspring neurodevelopment. Our results suggest that there is limited and controversial evidence for the protective effect of DHA intake on pregnancy complications, with the exception of preterm birth and gestational diabetes mellitus. However, additional DHA supplementation may improve long-term neurodevelopmental outcomes in the offspring of women with pregnancy complications.

## Introduction

Today, the world is experiencing a demographic transition towards long-term subreplacement fertility [1]. The low fertility rate is undoubtedly an important reason for the rapidly aging worldwide population [2], and this aging brings along a series of complex social problems [3]. Some countries have implemented policies to encourage childbearing, but these policies have resulted in an increase in advanced maternal age and the resultant high risk of pregnancy complications [4]. Therefore, increased attention to perinatal care, reduction in pregnancy complications, and improvement in adverse neonatal outcomes are the current goals of obstetricians and are essential for promoting social health and economic development.

To maintain normal physiological health, the body must intake various nutritional elements from food, especially glucose, fat and protein. Fat is the second-largest source of dietary energy for human beings [5], and fatty acids (FAs), which are obtained through the metabolism of fat, not only serve as energy sources but also play an important role in maintaining normal cellular physiology. Deficiencies or abnormal increases in FAs are associated with cardiovascular and neurodevelopmental diseases [6]. N-3 long-chain polyunsaturated fatty acids (n-3 PUFAs) are believed to play a central role in brain function and neuronal membrane structure, and are also necessary for the development of the myelin sheath and retina [7]. Approximately 90% of n-3 PUFAs in the brain are composed of docosahexaenoic acid (DHA) [8], suggesting the importance of DHA in maintaining brain function. In recent years, increasing epidemiological and clinical evidence has proven the preventive or therapeutic effects of DHA in Alzheimer’s disease [9], attention deficit and hyperactivity disorder [10], breast cancer [11], coronary heart disease [12] and some other diseases.

The importance of DHA is evident early in life. DHA is rapidly integrated into retinal and brain neural tissue during the last three months of pregnancy [13] and plays a significant role in early fetal neurodevelopment. Remarkably, the fetus and placenta rarely synthesize DHA autonomously [14]. Therefore, maternal DHA intake and placental transport function are critical for fetal DHA acquisition [15]. However, in some pregnancy complications, low maternal DHA levels [16, 17] or dysfunction in placental fatty acid transport [18, 19] leads to DHA deficiency in the offspring, which may lead to long-term neurological disorders. Thus, women with pregnancy complications may benefit from DHA supplementation. Additionally, healthy pregnant women and fetuses may also benefit from DHA supplementation due to its potential effects on neurodevelopment and its preventive effect on a variety of diseases [20]. However, there remain contradictions in the current relevant clinical studies. For example, Colombo et al. found that prenatal DHA may positively affect infants’ attention and regulation [21], whereas Gould et al. observed no benefit of prenatal DHA supplementation on child behavior; on the contrary, the results of Gould et al. suggest an adverse effect of DHA on behavioral functioning [22]. Clarifying the mechanism of action of DHA can help guide supplementation during pregnancy [23].

This review summarizes the research on the relationship between DHA intake during pregnancy and five common pregnancy complications: preeclampsia (PE), gestational diabetes mellitus (GDM), preterm birth (PT), intrauterine growth restriction (IUGR), and postpartum depression (PPD). Additionally, we discuss the impact of DHA intake during pregnancy on the prediction, prevention and treatment of the aforementioned pregnancy complications. Furthermore, we explore the role of DHA in offspring neurodevelopment. Finally, because DHA is the most important n-3 PUFA, this review also summarizes the relationship between n-3 PUFAs and the aforementioned pregnancy complications.

## Preeclampsia (PE)

Maternal plasma DHA levels are significantly altered in patients with PE. Dangat et al. collected peripheral blood from PE patients (n = 45) and normal pregnant women (n = 85) at the time of delivery and found that maternal plasma DHA concentrations were significantly reduced in PE patients [16], and similar results were reported in other studies [17]. In addition, women with the lowest n-3 PUFA levels in red blood cells were 7.6 times more prone to suffer from PE than those with the highest levels [24], but this conclusion may be confounded by causality. In fact, the decrease in plasma DHA levels in PE patients does not occur until the third trimester. A cross-sectional study suggested that the decrease in DHA in PE patients was already present at 16–20 weeks of gestation [18]. These results suggest that early maternal DHA levels may be potentially predictive of PE.

Previous studies have not found any difference in seafood intake between PE patients and normal pregnant women, suggesting that the decrease in plasma DHA content in PE patients is not caused by the decrease in maternal dietary intake. Mackay et al. proposed that the metabolic pattern of PE patients includes high nonesterified fatty acid (NEFA) concentrations [25]. Increased NEFA flux is associated with mitochondrial dysfunction and may cause ectopic lipid accumulation in the liver and other tissues, which interferes with PUFA synthesis [26]. Another explanation for the decrease in plasma DHA content in PE patients is oxidative stress in the placenta [27]. Reactive oxygen species attack the double bonds of PUFAs and initiate a chain reaction leading to the formation of lipid peroxides. There is a deficiency of antioxidants in PE, and the increased peroxidative reactions further promote the decomposition of PUFAs. Notably, Roy et al. found that maternal plasma DHA levels were lower in PE women who gave birth to male infants than in normal control pregnant women who gave birth to male infants, but this same relationship was not observed between PE and normal pregnant women who gave birth to female infants [28]. Taken together with in utero exposure to PE as an environmental risk factor for various neurodevelopmental disorders [29] and the role of DHA in neurodevelopment, this result suggests that male infants born to mothers with PE may be at higher risk for neurodevelopmental disorders than female infants.

In addition to maternal plasma, the DHA content in the cord blood of PE patients is also lower than that of normal pregnant women [18, 25, 30]. The decrease in cord blood DHA levels may be due to the following three reasons: (1) PE patients are in a low PUFA environment, which leads to a decrease in maternal plasma DHA content and a subsequent decrease in cord blood DHA content. (2) Increased oxidative stress in the placental tissues of patients with PE may lead to peroxidation of cord blood lipids. In addition, oxidative stress can also lead to the dysregulation of angiogenic factors, resulting in an increase in sFlt-1, which by itself has been shown to induce oxidative stress, leading to further decreases in the level of DHA in cord blood [30]. (3) Placental tissues of PE patients have been confirmed to have decreased mRNA levels of Δ5 desaturase and fatty acid transport protein 1/4 (FATP1/4) [18]. Unfortunately, Δ5 and Δ6 desaturases are the rate-limiting enzymes for PUFA conversion and are recognized as the primary determinants of PUFA levels, and the transport of DHA from mother to offspring is primarily carried out by FATP1/4 [31]. The decreased expression of FATP1/4 is a sign of impaired fatty acid transport in the placenta and a possible cause of decreased DHA content in cord blood. (4) Lipidomic analysis of placentas from patients with preeclampsia reveals higher lipid content than placentas from healthy patients [32]. Although the mechanism remains unclear, the presence of ectopic fat in the placenta suggests that DHA becomes trapped in the placenta and is not transferred to the fetus; thus, the amount of DHA in cord blood is low. The fatty acid transport family primarily includes fatty acid transport proteins (FATPs), fatty acid-binding proteins (FABPs) and fatty acid translocases (FAT/CD36) [33]. In human placental tissue, FABPs are predominantly located on the maternal-facing placental membranes, whereas FATPs and FAT are distributed across both maternal and fetal membranes [34]. FABP4 is essential for trophoblast lipid accumulation. Inhibition of FABP4 expression in primary human trophoblasts blocked their uptake of exogenous fatty acids [35]. In addition, Biron-Shental et al. found that human primary trophoblasts exhibited increased expression of FABP1, FABP3 and FABP4 under inflammatory and hypoxic conditions [36], and FABP4 expression was increased in both the serum and placenta of PE patients [37, 38]. Increased expression of FABPs located on the maternal surface and decreased expression of FATPs, which are primarily responsible for the transport of free fatty acids to the fetus, may be the cause of placental ectopic fat in patients with eclampsia. These results suggest that alterations in polyunsaturated fatty acid metabolism and transport in different regions of the preeclamptic placenta may contribute to the pathological features of preeclampsia.

**Fig. 1:**
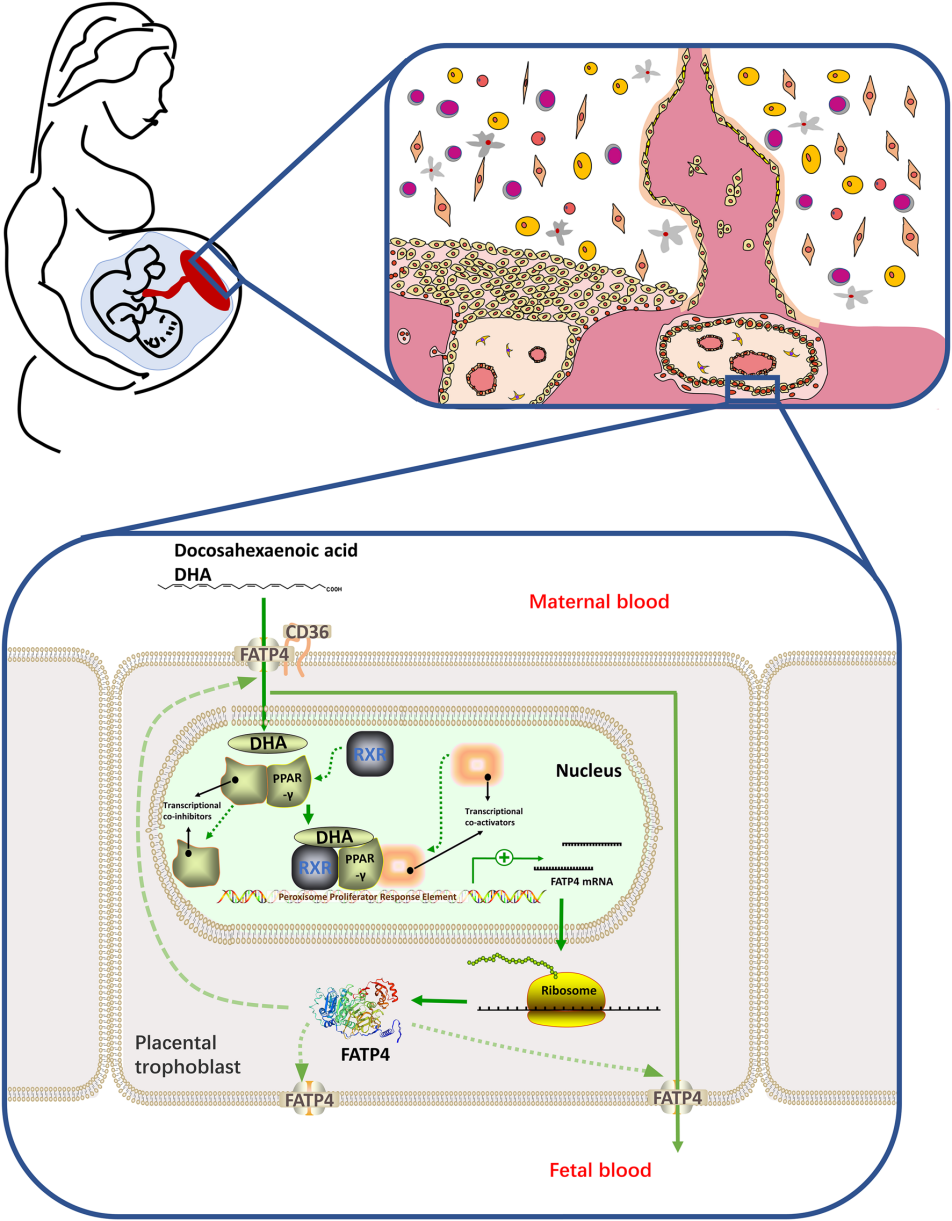
DHA upregulates FATP4 expression via PPAR-γ to promote its transport in the placenta

However, the decrease in cord blood DHA levels due to these three potential causes appears to be partially ameliorated by DHA supplementation during pregnancy. First, the low maternal plasma level of DHA in PE patients can be improved by exogenous DHA supplementation. Second, DHA can exert antioxidant effects by promoting mitochondrial function and biogenesis [39], which is speculated to antagonize oxidative stress in PE patients. Third, FATPs and FABPs are regulated by peroxisome proliferator-activated receptor γ (PPAR-γ) [40], whose natural ligand is primarily derived from dietary n-3 PUFAs, with DHA being the main component [41]. Therefore, DHA is expected to ameliorate the decreased expression of FATP1 and FATP4 in placental tissues of PE patients through the PPAR-γ pathway, subsequently promoting DHA transport, forming a process similar to “positive feedback” mechanism (Fig. 1). However, most of the current studies are observational studies, and no study has investigated the effects of DHA supplementation in PE patients on cord blood DHA content and long-term neurodevelopment in offspring. Future studies could use this as an entry point to explore the clinical value of DHA supplementation.

Interestingly, despite the decrease in maternal plasma DHA content in PE patients, the postpartum DHA content in breast milk increases [16, 42]. It is possible that when global PUFA levels are low in PE patients, there are adaptive mechanisms in the mammary gland to synthesize PUFAs by increasing sterol regulatory element binding proteins (SREBP-1), and the increase in SREBP-1 expression may ameliorate the expression of Δ5 and Δ6 desaturase [43]. This study seems to provide guidance for breastfeeding in PE patients. However, because there are few studies on this topic, further mechanistic studies and animal models are needed to explore the clinical significance.

A large number of epidemiological studies support the positive effects of n-3 PUFAs on cardiovascular events, including hypertension [44], which may be attributed to the antioxidant effect of n-3 PUFAs. The n-3 PUFAs can improve vascular endothelial function and antagonize the proinflammatory response in hypertension [45]. Meanwhile, n-3 PUFAs have also been shown to possess vasodilator effects [46]. Similar to hypertension patients, PE patients exhibit pathologic oxidative stress, inflammation and vasoconstriction. Therefore, n-3 PUFAs are also expected to improve the symptoms of PE patients. However, studies have reported conflicting results. A Cochrane study reported that PE may be reduced with omega-3 LCPUFAs (RR 0.84, 95% CI 0.69–1.01; 20 trials, 8306 participants; low-quality evidence) [47], whereas another RCT suggested that DHA supplementation of 800 mg/d in the second half of pregnancy does not reduce the risk of PE [48]. In contrast, Li et al. found that maternal DHA intake was inversely associated with the risk of PE, and dietary n-3 PUFA intake may protect pregnant women from the development of PE [49]. A Danish cohort study involving 65,220 pregnant women with singleton pregnancies suggested that although EPA + DHA 250 mg/d did not improve the overall risk of PE, supplementation reduced the risk of severe preeclampsia (RR = 0.77, 95% CI 0.60–0.99) [50]. In view of the current controversy about the risk and therapeutic effect of DHA on PE in pregnant women, we look forward to more large-scale RCTs on maternal DHA intake and PE risk to provide guidance on maternal DHA supplementation. Current studies primarily focus on whether the use of DHA in normal pregnant women can prevent PE and whether DHA supplementation in PE patients can improve abnormal blood pressure. Few studies have focused on the relationship between DHA supplementation in PE patients and long-term neurodevelopment in offspring. Future research could focus on this relationship.

In conclusion, PE patients exhibit lower plasma DHA concentrations, which have been observed as early as 16–20 weeks of gestation. Low plasma DHA levels, placental oxidative stress, the absence of fatty acid transporters, and ectopic fat in the placenta are potential reasons for the reduction in cord blood DHA concentrations in PE offspring. Whether supplementation with DHA during pregnancy can improve the reduction in cord blood DHA in eclamptic patients and the neurodevelopment of the offspring remains largely unknown and necessitates future research.

## Gestational diabetes mellitus (GDM)

In contrast to the decrease in maternal plasma DHA levels in PE patients, a cross-sectional study suggested that GDM did not affect the activities of Δ5 and Δ6 desaturases, and maternal plasma DHA levels were not decreased when GDM was diagnosed in the second trimester [51]. In a meta-analysis of 24 observational studies, plasma DHA levels were even elevated in GDM patients [52]. However, a high maternal DHA environment did not increase the DHA content in cord blood. Léveillé et al. did not identify any difference in DHA levels in cord blood between GDM patients and normal pregnant women [53], and even in most studies, DHA levels in the cord blood of GDM patients were found to be significantly lower than those of pregnant women with normal blood glucose [52, 54]. These results suggest that the ability of the GDM placenta to transport n-3 PUFAs like DHA is decreased.

Gázquez et al. found that DHA supplementation during pregnancy in normal pregnant women increased both maternal DHA levels and cord blood DHA levels simultaneously, but DHA supplementation in GDM mothers did not improve cord blood DHA levels [55, 56]. Studies of fatty acid placental transfer in vivo using stable isotope tracers have also confirmed the impairment of fatty acid transport in GDM placentas [19]. Previous studies have found that the expression of FATP1, FATP4, the Major Family Super Domain 2a (MFSD2a) and endothelial lipase in placental tissues of GDM patients is significantly decreased [57, 58], resulting in placental fatty acid transport disorders. MFSD2a is associated with the selective transportation of DHA as lysophospholipids. The expression of the DHA membrane transporter MFSD2a is lower in GDM placentas, which could affect maternal-fetal DHA transport. Therefore, the level of MFSD2a in the maternal blood of GDM mothers could be used as a potential biomarker for the early detection of disturbances in MFSD2a expression during pregnancy and the subsequent consequences on offspring neurodevelopment [57]. The fatty acid transport disorder in GDM placental tissue may be caused by environmental stimulation of high glucose and insulin resistance [59]. When GDM patients exhibit good blood glucose control, cord blood DHA levels are positively correlated with maternal DHA levels [60]. This result also explains why Léveillé et al. did not observe a reduction in DHA levels in the cord blood of GDM patients; the case group selected in this study was GDM patients with good blood glucose control by diet or insulin [53]. Therefore, controlling blood glucose levels through diet, exercise, and medications in GDM patients may facilitate placental fatty acid transport.

Decreased cord blood DHA levels in GDM patients may lead to decreased plasma DHA levels in GDM newborns [61, 62], and this result has been shown to affect neurodevelopment at 6 months after birth [63]. Because previous studies suggest that additional DHA supplementation did not significantly increase the DHA levels in cord blood [55, 56], how to improve GDM placental fatty acid transport disorders has become the focus of preventing neurodevelopmental disorders in GDM offspring. As mentioned previously, improving blood glucose by diet, exercise or medication may help restore normal fatty acid transport in the placenta. In addition, similar to the reduced expression of FATP1 and FATP4 in the placenta of PE patients, DHA is also expected to improve FATP1 and FATP4 expression and promote fatty acid transport through the PPAR-γ pathway in GDM (Fig. 1). Although DHA supplementation at 600 mg/d during pregnancy in GDM patients did not improve the status of fetal DHA [56], this dose was within the physiological recommended intake range, and the effects of higher doses of DHA supplementation on placental fatty acid transport function and umbilical cord blood DHA content cannot be determined. Given that n-3 PUFAs have been used at a dose of 2.4 g/d in overweight school-age children with metabolic syndrome [64] and have not been associated with adverse pregnancy outcomes including excessive weight gain in overweight pregnant women [65], future studies should evaluate the effect of higher doses of n-3 PUFAs on cord blood DHA levels in GDM patients under conditions of safety.

DHA levels in vivo are negatively correlated with several markers of insulin resistance [66]. Animal models have suggested that n-3 PUFAs may reduce the secretion of inflammatory cytokines and reverse glucose intolerance [67, 68]. Meanwhile, n-3 PUFAs can also improve pancreatic fatty infiltration in the offspring of GDM mice [69]. Therefore, DHA may also have some preventive and therapeutic effects on GDM. However, RCTs and cohort studies have demonstrated that supplementation of DHA-rich fish oil during pregnancy did not prevent the development of GDM in normal pregnant women [48, 66, 70], but pregnant women who have been diagnosed with GDM can reduce the levels of blood glucose, blood lipids, high-sensitivity C-reactive protein and insulin resistance by intake of cod liver oil [71]. Combined supplementation of vitamin D and n-3 PUFAs for 6 weeks in GDM patients has also been shown to have beneficial effects on fasting blood glucose, serum triglycerides, very low-density lipoprotein cholesterol, and insulin-related indicators [72]. Therefore, DHA plays a more therapeutic role than a preventive role in GDM patients. Patients with GDM during pregnancy may benefit from DHA supplementation, and DHA supplementation can be considered in the management of GDM patients during pregnancy. The clinical benefits of this initiative, such as whether it can control blood glucose levels in GDM patients and improve neurodevelopment in GDM offspring, remain to be evaluated in large-scale clinical trials.

In summary, although the plasma DHA level of GDM patients is unchanged or even increased, the umbilical cord blood DHA level is significantly decreased. These results suggest that placental transport dysfunction in GDM, which has been verified in many other studies, may be the primary cause of reduced DHA uptake in GDM offspring. High-dose DHA may improve the reduction in cord blood DHA in GDM patients. In addition, according to existing studies, although DHA supplementation during pregnancy does not seem to prevent GDM, pregnant women with GDM may benefit from DHA supplementation during pregnancy.

## Preterm birth (PT)

A case‒control design nested in the Danish National Birth Cohort measured the percentage of plasma DHA + EPA in total fatty acids at 9 and 25 weeks of gestation in 376 pregnant women with early preterm birth (< 34 weeks, ePT) and 348 pregnant women with term pregnancy [73]. Compared with women with DHA + EPA concentrations ≥ 1.8%, women with concentrations < 1.6% had a 10.27-fold increased risk of ePT. This result suggests that low plasma DHA and EPA concentrations during pregnancy may be a strong risk factor for preterm birth. A cross-sectional study in 2021 also suggested that the plasma DHA levels of PT pregnant women at delivery were significantly lower than those of full-term pregnant women [17]. However, this study did not compare the plasma DHA levels of PT pregnant women at delivery with the plasma DHA levels of full-term pregnant women at the corresponding gestational weeks, and there is no study to dynamically monitor the changes in plasma DHA levels of pregnant women throughout the whole pregnancy, so it is not possible to confirm whether the results are caused by different gestational weeks. In general, the low plasma DHA levels during pregnancy seem suggestive of a relationship with PT [74], and some scholars have advocated monitoring the plasma concentration of DHA + EPA during pregnancy to predict PT [75].

The mechanism by which DHA levels contribute to PT remains unclear, although several have been suggested: (1) The initiation of labor is associated with increased expression of uterotonic proteins, activation of specific ion channels, and increased connexin 43 [76]. These factors promote electrical synchronization and coordination of contractions in the myometrium. Upon activation of contractile protein receptors, the uterus can be stimulated to contract by oxytocin and the stimulatory prostaglandins E2 and F2α. As one of the n-3 PUFAs, the physical properties of the lipid membrane of DHA may affect the activity of contractile protein receptors [77]. In addition, DHA regulates connexin 43 expression [78] and reduces PGE2 and F2α levels [79]. (2) The infiltration of white blood cells and the release of cytokines are the triggers for the initiation of labor, and this maternal inflammatory response provides a protective effect for mother and baby [80, 81]. However, abnormal inflammation is considered the cause of PT [82], and DHA can reduce inflammation by regulating the interaction between ligands and receptors on the cell surface [83]. The lack of DHA in the third trimester of pregnancy may lead to abnormal activation of the inflammatory response and subsequent PT. This theory has been supported by clinical studies in which DHA supplementation during pregnancy contributes to the prolongation of gestational age [84]. (3) Defective deep placentation, a failure of invasion and transformation of the spiral arteries by the trophoblast, may cause uteroplacental ischemia, which is one of the causes of PT [85]. Studies of fatty acids on trophoblast cells have demonstrated that DHA has a proangiogenic effect, stimulating the production of proangiogenic factors [86] and improving the development of capillary buds [87]. Therefore, DHA may reduce the incidence of preterm birth by improving deep placental dysfunction.

Considering the above three reasons, it is speculated that DHA supplementation during pregnancy is a potential way to prevent PT. In 2018, the Cochrane study included 70 RCTs to analyze the association between n-3 PUFA supplementation during pregnancy and pregnancy outcomes, and found that pregnant women with n-3 PUFA supplementation during pregnancy exhibited reduced incidences of PT and ePT compared with those without DHA supplementation [47]. Three RCTs since 2018 have also suggested that DHA supplementation during pregnancy can reduce the risk of PT [88–90]. Among them, Carlson et al. found that daily supplementation with 1000 mg DHA was more effective in preventing ePT than daily supplementation with 200 mg DHA, and the effect was more significant in pregnant women with low baseline DHA levels [89]. Although these studies suggest the preventive significance of DHA supplementation during pregnancy for PT, further studies are needed to explore the relationship between maternal DHA levels and appropriate DHA intake; women with higher n-3 PUFA levels content have a lower risk of PT, and it is possible that additional supplementation with n-3 PUFAs may actually increase the risk of PT [91].

Moreover, the accumulation of fetal n-3 PUFAs primarily occurs during the last trimester of pregnancy [92], with the efficiency of n-3 PUFA transport from mother to fetus reaching a peak of 42–75 mg/d at 35–40 weeks of gestation [93]. Due to the early end of this period, preterm infants are especially prone to lack DHA in the critical window period of neurodevelopment [94], which is more likely to cause adverse outcomes, including long-term neurodevelopmental disorders in ePT [95]. Therefore, it is important and difficult to improve the low n-3 PUFA status of preterm infants. Human milk is the preferred source of nutrition for premature infants when they are able to receive enteral feeding, but the DHA content in breast milk is generally low due to the changes in modern dietary structure and the decrease in fish consumption [94, 96]. Adding to the problem, Vizzari et al. found that DHA content in breast milk appears to be related to gestational age, with breastmilk from preterm women having lower DHA content than that from full-term women [97]. These studies suggest that preterm infants who do not absorb enough DHA in the third trimester may benefit from DHA supplementation of the infants and their nursing mothers. Therefore, many scholars have advocated increasing the content of DHA in formula milk powder or food for preterm infants [97–99]. However, there is significant heterogeneity in related studies on this topic, and whether preterm infants can benefit from DHA supplementation is still inconclusive. Regarding offspring neurodevelopment, Hewawasam et al. found that DHA supplementation in preterm infants did not improve the attention of the offspring at 18 months [100], and the Cochrane analysis of the relationship between preterm infants and fatty acid supplementation did not find long-term benefits or harms in preterm infants receiving n-3 PUFA formula milk powder [101]. However, the study by Westerberg et al. suggested that early DHA supplementation in very low birth weight newborns had a positive effect on the attention ability of offspring at 20 months of age [102]. Moreover, DHA supplementation in preterm infants may reduce the risk of hay fever [103], necrotizing enterocolitis [104], and intraventricular hemorrhage [105].

In general, low plasma DHA levels during pregnancy seem to be suggestive of PT risk, and DHA supplementation during pregnancy has a preventive effect on PT and ePT. Premature infants have insufficient intake of DHA due to the early termination of pregnancy. There is considerable controversy regarding whether preterm infants benefit from postnatal DHA supplementation. RCTs with large sample sizes are needed to determine the clinical significance of DHA supplementation in preterm infants.

## Intrauterine growth retardation (IUGR)

Although DHA has been shown to be closely related to fetal neurodevelopment, there are few studies on the relationship between DHA and IUGR. A previous study found that DHA levels in maternal plasma and umbilical cord blood of pregnant women with IUGR fetuses decreased during pregnancy, whereas the placental expression of lipoprotein lipase, FABP1/3 and FATP1/2/4 increased [106]. The increase in placental fatty acid transporters may be a compensatory response, but this compensation did not improve the DHA deficiency of IUGR fetuses. It is not known whether the decrease in maternal DHA levels is a cause or consequence of IUGR. Although placental disorders are also predisposing factor for IUGR (see preterm birth section) [85], a meta-analysis suggested that n-3 PUFA supplementation during pregnancy did not reduce the risk of IUGR [47].

Given that the major site of action of DHA in the fetal period is the brain, two studies have evaluated the relationship between DHA supplementation during pregnancy and fetal head circumference [107, 108]. However, the two studies differed in their results, despite using the same DHA dose and similar start and end times. A Mexican study demonstrated that maternal intake of 400 mg/d DHA starting at 20 weeks of gestation was associated with a larger head circumference at birth [108], whereas another study suggested that intake of 400 mg/d DHA starting before 20 weeks of gestation until delivery did not increase the head circumference [107]. Gamboa et al. found that inactivating mutations in MFSD2A, required for n-3 PUFA transport in the brain, cause lethal microcephaly syndrome [109]. This result suggests that DHA deficiency may contribute to microcephaly. If clinical studies confirm this theory, DHA supplementation may be a potential treatment for women with microcephaly or small head circumference detected by ultrasound during pregnancy.

Notably, some animal models suggest that offspring with IUGR may benefit from maternal DHA supplementation, such as enhanced lung function [110, 111] and prevention of impaired oligodendrogenesis [112]. In addition, DHA supplementation in the offspring of IUGR model rats can improve cognition [113].

In conclusion, plasma and cord blood DHA levels are decreased in IUGR patients. DHA supplementation during pregnancy may have a protective effect on fetal microcephaly and small head circumference. Despite the demonstrated benefits of maternal and offspring DHA supplementation for IUGR infants in animal models, there are substantial gaps in relevant clinical studies, which should be the focus of future research.

## Postpartum depression (PPD)

Previous studies have suggested that EPA, DHA and total n-3 PUFA levels are lower in patients with depression, suggesting their role in the pathogenesis of depression [114, 115]. Related RCTs and meta-analyses also suggest that n-3 PUFAs may exhibit preventive and therapeutic effects on depression [116–118] through their role in anti-inflammatory, antioxidant, and neurotrophic processes in the brain [119]. Similar to depression, patients who develop PPD have been shown to exhibit low levels of DHA in the postpartum period [120], which may occur in the third trimester [121] or even the first trimester [122]. Epidemiological evidence suggests that both higher DHA concentrations in breast milk and higher seafood consumption predict a lower prevalence of PPD [123], and greater seafood consumption and n-3 PUFA intake may be protective against PPD [124]. This evidence suggests the potential predictive, preventive and therapeutic effects of n-3 PUFAs in PPD.

However, the results of RCTs have been more divergent. A large RCT involving 2399 pregnant women suggested that DHA supplementation at 800 mg daily starting at 21 weeks of gestation did not reduce maternal PPD risk [125]. Other studies have reached similar conclusions [126–130]. For pregnant women with low DHA levels during pregnancy, additional DHA supplementation did not seem to prevent PPD [131]. DHA supplementation of 1.9 g/d for 8 weeks also did not exhibit a significant therapeutic effect on women who already developed PPD [130], and the result of Mendelian randomization did not demonstrate a causal relationship between n-3 PUFA intake and PPD [132]. In contrast, several recent studies have suggested an association between maternal DHA levels and PPD [133], as well as the preventive or therapeutic effects of DHA supplementation on PPD [134, 135]. We compared the differences between different studies and found that the year of study was the most significant factor explaining the differences in conclusions. Most of the negative studies [125–132] were conducted relatively early, with even the most recent negative studies [126, 127] published in 2017. However, most of the positive studies [133–135] were conducted in recent years, all of which were performed after 2020. The specific effect of study years on this outcome is not clear. Changes in social environment and dietary structure may be potential causes, but more clinical studies are needed to verify the results. The two recently published meta-analyses [136, 137] were limited by the lack of comprehensiveness of the included studies, and the conclusions were inconsistent, which had limited the creation of clinical guidelines. Although Cochrane did not conclude a protective effect of n-3 PUFAs against PPD [138], this meta-analysis is a decade old. Therefore, it is necessary to conduct a systematic, comprehensive and rigorous analysis of the studies on the association between n-3 PUFA intake and PPD.

In addition, because one-third of women with PPD experience the onset of depressive symptoms during pregnancy [139], we also summarized the effects of DHA intake on depression during pregnancy. Epidemiology suggests an association between low omega-3 intake from seafood and increased risk of elevated depressive symptoms during pregnancy [140], and the intake of fish and DHA has a protective effect on depression during pregnancy [141]. Rees et al. found that women with depression during pregnancy have lower blood DHA levels in the third trimester [142]. After adjustment for confounding factors, those with high DHA levels still had significantly lower odds of depression. Another prospective cohort also found that lower DHA serum concentrations, regardless of the pregnancy trimester in which levels were measured, were associated with higher odds of depressive symptoms throughout pregnancy [143]. Depressive symptoms in early pregnancy, even in the subclinical range, were inversely associated with breast milk DHA levels [144], which may have some impact on the offspring. Given that DHA appears to have a protective effect on depression during pregnancy [145], women with depressive symptoms may benefit from DHA supplementation during pregnancy, both for themselves and their offspring.

In summary, similar to patients with depression, DHA levels were decreased in PPD patients. Epidemiological evidence suggests that DHA supplementation has a potential role in the prediction, prevention and treatment of PPD. There is a large variation in clinical studies, and the year of study, which may reflect social development, dietary structure improvements and changes in the preparation of DHA, may be the main reason for this variation. In addition, DHA may also have a protective effect on depression during pregnancy.

## Conclusions and future perspectives

Although significant evidence suggests that DHA supplementation during pregnancy may aid in the prevention of PT and may represent a treatment for GDM, in the data is less clear with respect to the prevention or treatment of other pregnancy complications. In view of the suggestive role of maternal DHA levels in these diseases, and the finding that only pregnant women with low maternal DHA levels could benefit from additional DHA supplementation in some studies, we suggest that the measurement of maternal DHA levels during pregnancy may have a positive effect on maternal perinatal management.

Studies suggest that a daily intake of 200 mg DHA can decrease the likelihood of preterm birth [146], and the recommended daily intake of DHA for pregnant women in many countries meets this requirement [89, 147]. Although studies suggest that 1000 mg/d DHA is more effective than 200 mg/d DHA in preventing ePT [89], there are few studies on the dose, and this relationship seems to be influenced by maternal DHA levels [91]. Therefore, we do not recommend DHA supplementation in healthy pregnant women beyond the doses recommended in the dietary guidelines because it does not appear to provide additional benefit. Unfortunately, in modern diets, the intake of fish containing high levels of n-3 PUFAs is limited [83]. In the United States, the average adult daily intake of DHA + EPA is approximately 100 mg [148], well below the recommended daily intake of DHA during pregnancy. Therefore, when normal pregnant women are unable to meet the appropriate intake of DHA, additional supplementation with DHA capsule preparations has a positive effect on pregnancy outcomes and fetal development.

Whether DHA supplementation should be added to the recommended intake of 200 mg/d for pregnant women with comorbidities remains questionable. The prevention and treatment effects of DHA for pregnancy complications are controversial, and there is a lack of related studies on the long-term neurodevelopment of offspring of women with complications. Previous animal studies have found that the water maze performance of healthy young mice fed DHA until adulthood is not different from that of healthy mice fed without DHA supplementation [149]. However, in animal models such as Alzheimer’s disease [150], traumatic brain injury [151], n-3 PUFA deficiency [152, 153] and offspring exposed to general anesthesia with propofol during early pregnancy [154], water maze related performances were significantly improved after DHA supplementation. In view of this phenomenon, we believe that the effects of DHA on nerve development and wound repair may only be reflected in pathological conditions. For women with normal pregnancies who have normal levels of DHA and meet the daily requirements for DHA intake, additional DHA supplementation may not confer benefit in the offspring. However, for mothers with certain pregnancy complications, even if their DHA levels are normal or they consume the recommended amount of DHA daily, the offspring may still be exposed to low DHA levels due to placental transport disorders, which may lead to long-term neurodevelopmental disorders. In such cases, although high-dose DHA supplementation may not play a role in the treatment of complications, it may improve DHA deficiency in offspring by increasing maternal DHA levels, improving placental fatty acid transport, and preventing the long-term neurodevelopmental disorders caused by some pregnancy complications.

Current studies on DHA and offspring neurodevelopment primarily focus on healthy pregnant women without complications. We hope that future studies can explore the effects of DHA supplementation on the long-term neurodevelopment of offspring in complicated pregnancies, and provide new guidance for the necessity of DHA supplementation in women with complicated pregnancies.

## Data Availability

Not applicable.
